# Difficulties of Management of Multiple Synchronous Bone Tumors in Li-Fraumeni Syndrome

**DOI:** 10.1155/2019/8732089

**Published:** 2019-11-13

**Authors:** Marine Huby, Laurence Brugières, Eric Mascard, Nathalie Gaspar, Stéphanie Pannier, Jean-Charles Aurégan

**Affiliations:** ^1^Department of Pediatric Orthopedics, Necker-Enfants Malades Hospital, AP-HP, University Paris Descartes, 149 Rue de Sèvres, 75014 Paris, France; ^2^Department of Pediatric and Adolescents Oncology, Gustave Roussy Cancer Campus, Paris Saclay-University, 114 Rue Édouard Vaillant, 94800 Villejuif, France

## Abstract

Li-Fraumeni syndrome is a rare inherited disease characterized by the early onset of multiple primary malignant tumors. Sarcomas account for more than 30% of all malignant tumors occurring at pediatric age. Furthermore, it was shown that the rates of second cancer were higher in childhood cancer survivors. We report the case of a patient with Li-Fraumeni syndrome who was referred to us with three synchronous skeletal tumors. This unique situation led to difficulties for the medical team regarding the diagnosis of malignancy and the surgical treatment to propose. The discovery of multiple lesions in the extension assessment underlines the usefulness of whole-body imaging for the follow-up of patients with germline *TP53* mutations. Most recent guidelines now recommend annual whole-body MRI for screening for cancer patients carrying germline TP53. With this report, we aim to share our experience with this rare situation in order to improve care about these specific cases.

## 1. Introduction

Li-Fraumeni syndrome (LFS) is a rare inherited disease characterized by the early onset of multiple primary malignant tumors. Li and Fraumeni described it in 1969 [1]. The initial diagnosis criteria of LFS were the occurrence of a sarcoma under the age of 45 years, associated with a first-degree relative who had any cancer before 45 years of age, and a first- or second-degree relative who had any cancer before 45 years of age or a sarcoma at any age [2]. New criteria were proposed in order to integrate the different clinical situations suggestive of LFS: (1) a proband with a tumor belonging to the narrow LFS tumor spectrum (soft tissue sarcoma, osteosarcoma, brain tumor, premenopausal breast cancer, and adrenocortical carcinoma) before 46 years and at least one first- or second-degree relative with a narrow LFS tumor (except breast cancer if the proband is affected by breast cancer) before 56 years or with multiple tumors; or (2) a proband with multiple tumors (except multiple breast tumors), two of which belong to the narrow LFS tumor spectrum and the first of which occurred before 46 years; or (3) patient with adrenocortical carcinoma, choroid plexus tumor, and rhabdomyosarcoma of embryonal anaplastic subtype irrespective of the family history; and (4) breast cancer before 31 years [3–5].

In 1990, Malkin et al. described the role of the germline mutation of the tumor suppressor gene *TP53* in LFS families [6]. Later, Varley found that this mutation of *TP53* was found in 77% of families with LFS [7]. Pathogenic *TP53* mutations contribute to a high risk of developing multiple cancers. In a report from the French group, more than 40% of 322 mutation carriers developed multiple tumors, either synchronous or metachronous [5]. The respective role of the genetic background and of cytotoxic therapy (chemotherapy and/or radiotherapy) in the occurrence of these secondary malignancies is not well known yet. Furthermore, the rate of second cancer was shown to be higher in childhood cancer survivors [8, 9].

We present the case of an LFS patient referred to us with three synchronous skeletal tumors. We will discuss the clinical presentation at diagnosis, the treatment, and the outcome at last follow-up.

## 2. Case Report

A seventeen-year-old girl was referred for subacute foot pain. In her past medical history, she presented with rhabdomyosarcoma of the left thoracic wall at the age of 8 months treated by surgery and chemotherapy. In her family medical history, her mother developed breast cancer and a leiomyosarcoma diagnosed at the age of 45, two of her maternal aunts in her mother's side were diagnosed for breast cancer, and one of her first-degree cousins with a rhabdomyosarcoma. The genetic analysis found a *TP53* mutation c673-2A>G at the splice acceptor site of intron 6.

**Figure 1:**
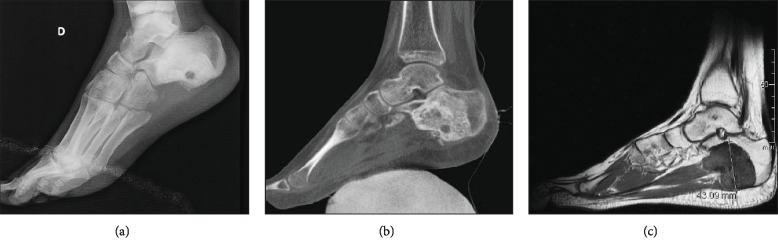
Osteosarcoma of the right calcaneus. (a) Lateral radiographs of the ankle. (b) CT scan of the right foot (sagittal). (c) MRI T1 FSE sagittal.

**Figure 2:**
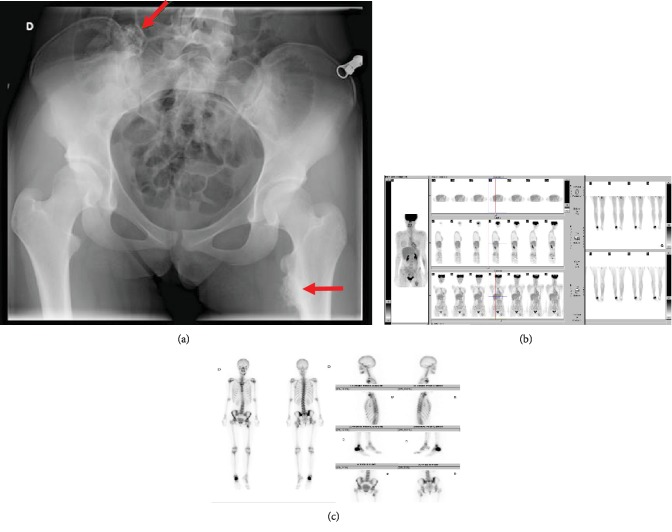
Surveillance of the tumor of the right calcaneus. (a) Initial anteroposterior radiograph of the pelvis. (b) TEP scan of the right calcaneus (SUVmax 7), upper metaphysis of the femur (SUVmax 2.8), and right sacroiliac (SUVmax 1.9). (c) Bone scintigraphy: intense hyperfixation of the right calcaneus, moderate hyper fixation of the lesser trochanter, and low fixation of the right sacroiliac articulation.

Her recent history was marked by the occurrence of a continuous pain on the medial aspect of her right foot. Plain radiographs of the ankle and the foot revealed an ossification of the calcaneus (Figure 1(a)). The presence of a tumor was confirmed by a CT scan (Figure 1(b)) and a MRI (Figure 1(c)). The biopsy revealed a high-grade osteosarcoma of the right calcaneus. On the PET scan, three different hypermetabolic lesions were seen: one intense hyper fixation of the right calcaneus (maximum of standardized uptake values SUVmax 7), one moderate hyperfixation of the upper metaphysis of the femur (SUVmax 2.8), and one low hyperfixation of the right sacroiliac (SUVmax 1.9) (Figure 2(a)). These results were confirmed by scintigraphy (Figure 2(b)). Based on the radiological aspect and low FDG uptake, the two lesions incidentally discovered were diagnosed as osteochondromas (Figure 2(c)). It was decided to treat the high-grade osteosarcoma of the right calcaneus and to resect the two lesions after the end of chemotherapy.

**Figure 3:**
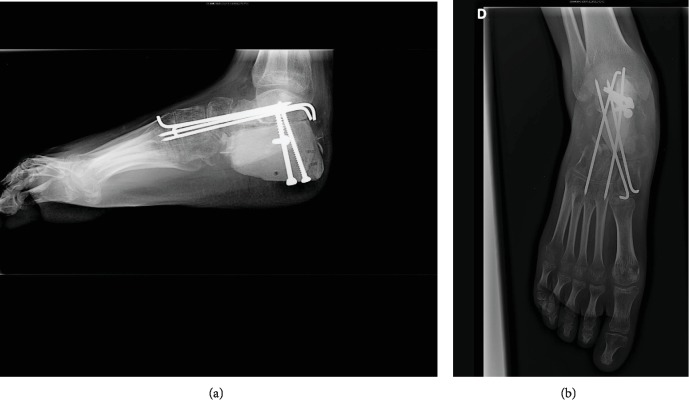
Lateral (a) and anteroposterior (b) radiographs of the right calcaneus. Postoperative result at 3 months of the resection of the right calcaneus with a reconstruction by iliac graft along with a triple arthrodesis of the hindfoot.

The initial course of treatment of the high-grade osteosarcoma of the right calcaneus was chemotherapy, and then, the multidisciplinary team proposed a conservative surgery. A resection of the right calcaneus with a reconstruction by iliac graft along with a triple arthrodesis of the hindfoot was performed (Figures 3(a) and 3(b)). The histopathology analysis of the tumor revealed a good response to chemotherapy with less than 5% of residual tumor cells identifiable. The postoperative period was marked by a deep infection of the operative site during chemotherapy requiring surgical treatment with removing the hardware along with the bone graft and adapted antibiotherapy.

**Figure 4:**
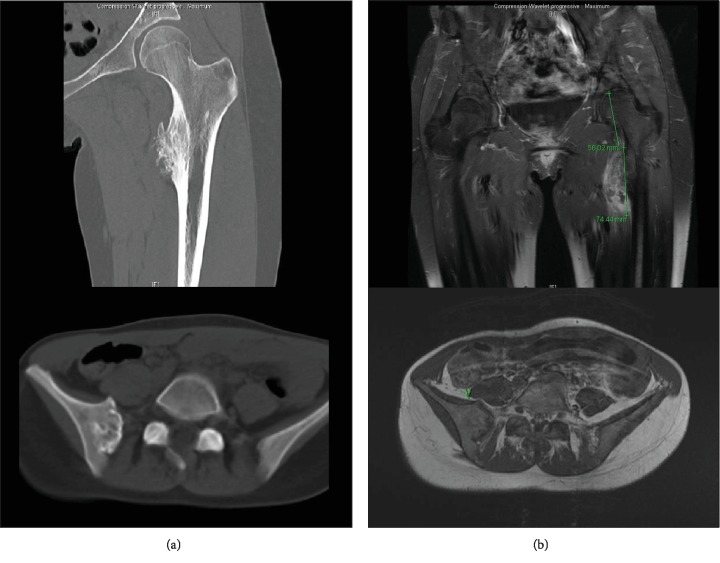
Lesions of the right sacroiliac and of the lesser trochanter: (a) aspect on CT scan and (b) aspect on MRI T1 Gado FAT SAT.

**Figure 5:**
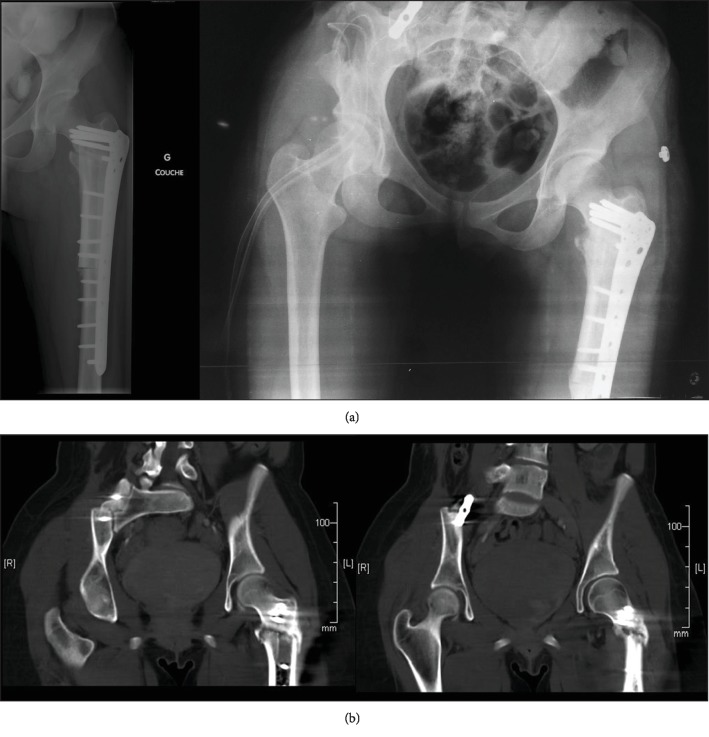
Postoperative imaging of the proximal left femur and of the right sacroiliac joint: (a) standard anteroposterior radiographs and (b) coronal CT scan.

The two lesions identified on the PET scan were followed up. CT and MRI performed 7 months after the end of chemotherapy showed only a thickening of the femur tumor's cap while the aspect of the sacroiliac lesion stayed stable (Figures 4(a) and 4(b)). In addition to these findings, the patient began to feel pain at the left hip. Then, it was decided to resect both tumors with large resection margins. The surgery of the proximal left femur, performed 2 years after the discovery of the lesions, was a resection of the left proximal femur with a reconstruction by allograft and fibula autograft (Figure 5(a)). The histopathological analysis of the tumor revealed that it was, in fact, a high-grade surface osteosarcoma. Finally, nine months later, the resection of the tumor of the right sacroiliac joint was performed. The sacral vertebras were conserved, and arthrodesis of the right sacroiliac joint was performed (Figure 5(b)). The histopathological analysis revealed a well-differentiated surface chondroblastic sarcoma. In both cases, the resection was complete, so no adjuvant treatment has been added.

At the last follow-up of eight years, no recurrence had occurred, and the patient was pain-free with an unlimited walking capacity. She was submitted then to annual screening including whole-body MRI and was diagnosed with 3 new tumors (a breast carcinoma at age 28, a melanoma at age 30, and a soft tissue sarcoma at age 31).

## 3. Discussion

Osteosarcomas are malignant tumors regularly reported among LFS patients [5]. It has been shown that 9.5% of young osteosarcoma cases (age < 30 years) carry a known pathogenic *TP53* mutation (3.8%) or a rare exonic *TP53* variant (5.7%) whereas none were identified in patients aged 30 years and older [10]. Few cases of metachronous osteosarcomas in LFS patients have been reported [11, 12]. However, the situation of three synchronous bone tumors is unique and raises questions about the best course of treatment.

The discovery of two additional lesions in the extension assessment underlines the need for whole-body imaging in patients with germline *TP53* mutations. Because of the broad spectrum of cancer associated with germline *TP53* mutations, cancer screening is challenging in this context. Since a few years, several reports have shown that whole-body MRI (WBMRI) allowing to detect cancers while still asymptomatic was efficient in the context of Li-Fraumeni syndrome. Indeed, this technique has been proven feasible, and several research protocols have demonstrated the efficacy of protocols including this procedure [13, 14]. A meta-analysis of the results of WBMRI in patients with Li-Fraumeni estimated that this procedure enables to detect cancer in 7% of asymptomatic mutation carriers at baseline. The international cancer screening recommendations for individuals with Li-Fraumeni syndrome published in 2017 recommend annual WBMRI alongside with regular clinical examination, regular abdominopelvic ultrasound and brain MRI in children, breast MRI, brain MRI, and dermatologic examination and digestive endoscopy in adults [15]. A substantial proportion of tumors detected by surveillance are low grade or premalignant. Given the risk of transformation in this context, early identification and management of such lesions is recommended [13].

In the case of our patient, the two additional lesions presented several criteria of benignity: no pain, no evolution during the follow-up, no mass in the soft tissues, no cortical lysis, and a very low hyperfixation in both bone scintigraphy and PET scan. Only one pejorative aspect was noted: the axial localization in the pelvis and proximal femur [16]. However, because a patient with a *TP53* mutation presents a high risk to developing cancer—cumulative incidence of cancer of 50% by age 31 in females and by age 46 in males and nearly 100% in both sexes at age 70—and because the usual criteria used to differentiate benign and malignant tumors are based on a low level of certitude, every tumor should be suspected to be malignant until proven otherwise in a patient with a germline *TP53* mutation [9, 17, 18].

For this patient, the rigorous follow-up allowed a curative treatment. Prognosis of osteosarcoma was dramatically improved by the introduction of chemotherapy and achieved 60-70% success compared to 10-20% with surgery alone. Bacci et al. studied the prognosis of synchronous multifocal osteosarcoma accounting for 3.6% of all patients with osteosarcoma treated in their institution (43/1154 patients). Only 16 patients were eligible for surgical treatment of all the tumors, 15 had complete remission, but only three remained free of disease after 5 years. This study showed the poor prognosis of this type of tumor. Furthermore, they suggested that they were bone-to-bone metastases of a single tumor because the response to chemotherapy was concordant in 13 of 16 cases [19]. In contrast, the case we present had two different histological types of osteosarcoma and two different responses to chemotherapy: good for the right calcaneus whereas there was no sign of necrosis in the lesion of the left femur resected 2 years after the initial diagnosis.

Finally, it is also possible that LFS could have favored the occurrence of a malignant tumor on a preexistent benign tumor. Chondrosarcoma is the second most common bone tumor after osteosarcoma, representing almost 20% of all bone tumors [20]. Mutations of *TP53* have been found in a minority of chondrosarcoma, and when present, it seems to be associated with a higher grade of tumor [21]. Secondary chondrosarcoma develops from a preexistent cartilaginous lesion, usually an enchondroma or osteochondroma. The estimated risk of secondary chondrosarcoma is about 1% in solitary osteochondroma and 5% in hereditary multiple exostoses (HME). According to these results, a study in Washington State estimated that the risk of developing chondrosarcoma in HME is increased by 1000 to 2500 over the risk of patients without HME [22]. Hence, LFS may have favored the development of secondary chondrosarcoma because of the presence of the *TP53* mutation.

## 4. Conclusion

The discovery of multiple lesions in the extension assessment raises the problem of the surveillance of patients with germline *TP53* mutations. Because of the broad spectrum of cancer associated with germline *TP53* mutations, cancer screening is challenging in this context. Since a few years, several reports have shown that whole-body MRI allowing to detect cancers while still asymptomatic was efficient in the context of Li-Fraumeni syndrome. With this report, we aim to share our experience of this rare situation in order to improve care about these specific cases.
